
JOURNAL INFORMATION
==============================
NLM Title Abbreviation: Environ Microbiol Rep
Journal ID: Environ Microbiol Rep
NLM Journal ID: 101499207
Journal ID: EMI4

Environmental Microbiology Reports

EISSN: 1758-2229
Publisher: Wiley

ARTICLE INFORMATION
==============================
PMCID: PMC10464663
DOI: 10.1111/1758-2229.12967
Article ID: EMI412967
Article version: 1
Subjects: Issue Information, Issue Information

Issue Information

Electronic publication date: 2023 May 5
Publication date: 2023 Jun
Volume: 15
Issue: 3
First page: 155
Last page: 156
Copyright: © 2023 The Author. Environmental Microbiology Reports published by Applied Microbiology International and John Wiley & Sons Ltd.
License: This is an open access article under the terms of the http://creativecommons.org/licenses/by/4.0/ License, which permits use, distribution and reproduction in any medium, provided the original work is properly cited.
License URL: https://creativecommons.org/licenses/by/4.0/

Figure count: 0
Table count: 0
Page count: 1

source-schema-version-number: 2.0
cover-date: June 2023
details-of-publishers-convertor: Converter:WILEY_ML3GV2_TO_JATSPMC version:6.3.3 mode:remove_FC converted:29.08.2023

==============================

VOLUME 15 NUMBER 3 JUNE 2023 www.env-micro.com ISSN 1758-2229

### ENVIRONMENTAL MICROBIOLOGY REPORTS

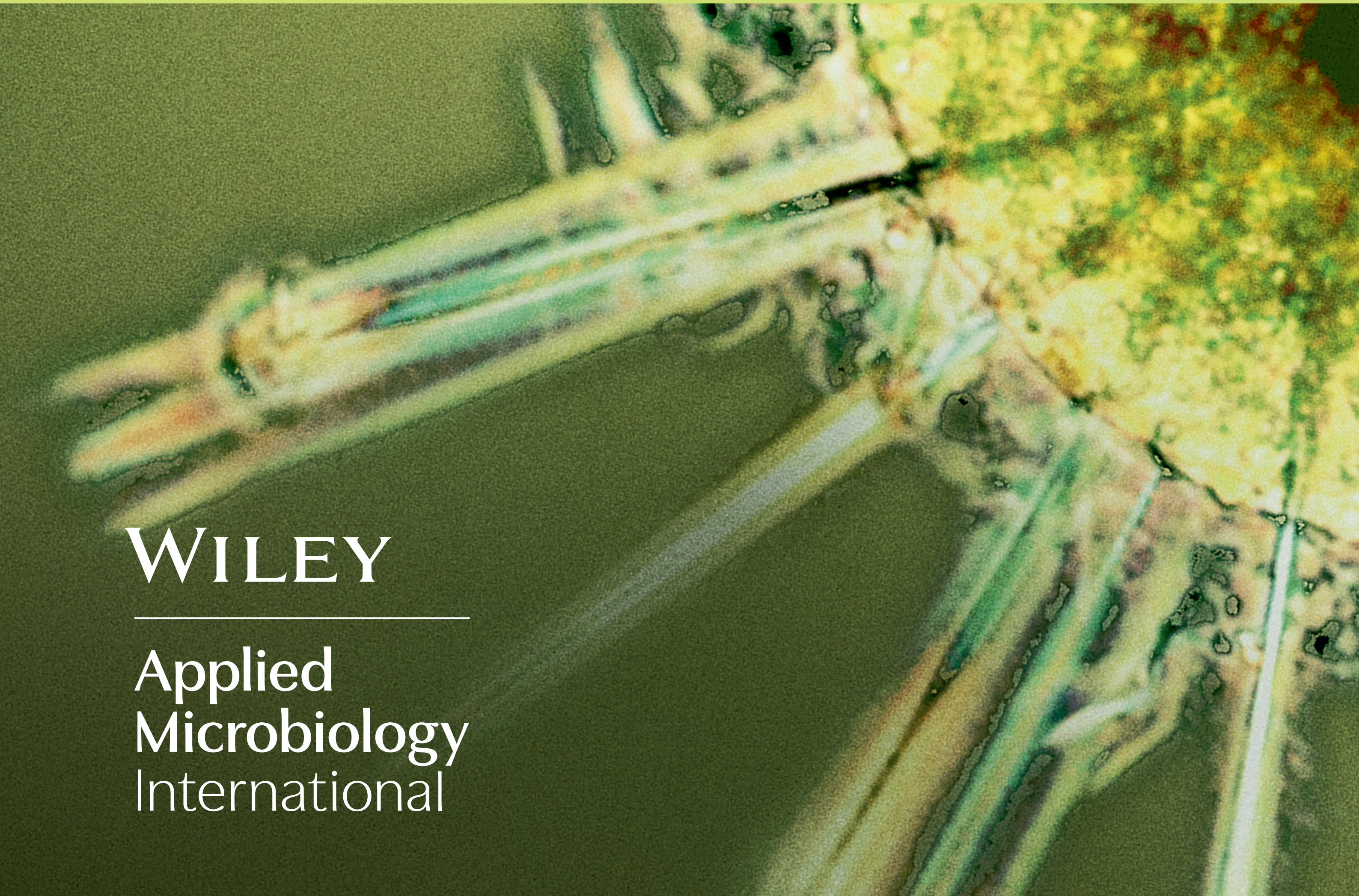

WILEY

Applied Microbiology International environmental
microbiology reports

### EDITORS

**Lukasz Grzeskowiak** Editor-in-Chief, Wiley.
Email: lgrzeskowi@wiley.com

**Maureen Coleman** University of Chicago, Department of the Geophysical Sciences, 5734 S. Ellis Ave., Chicago, IL 60637, USA.
Tel. +1-617-519-9396.
Email: mlcoleman@uchicago.edu

**Michael Cunliffe** The Marine Biological Association, The Laboratory, Citadel Hill, Plymouth, Devon,
Email: micnli@mba.ac.uk

**Antoine Danchin** Robustness and Evolvability of Life, Department of Infection, Immunity and Inflammation, Institut Cochin, INSERM U1016 - CNRS UMR8104 - Université Paris Descartes, 24 rue du Faubourg Saint-Jaqcues, 6th floor, 75014 Paris, France.
Tel: +33 (0) 1 44 41 25 51.
Email: antoine.danchin@normalesup.org

**Wei Huang** Department of Engineering Science, University of Oxford, UK.
Email: wei.huang@eng.ox.ac.uk

**Trina McMahon** University of Wisconsin, Madison, WI 53706-1691. Tel: +1 608-609-7814.
Email: kdmcmahon@wisc.edu

**Frank Stewart** School of Biology, Georgia Institute of Technology, Ford ES&T Building, Office 1242, 311 Ferst Drive, Atlanta, GA 30332-0230,USA.
Tel: 404-894-5819.
Email: frank.stewart@biology.gatech.edu

### GENOMICS UPDATES EDITOR

**Michael Galperin**, National Center for Biotechnology Information, NLM, National Institutes of Health, Bldg 38A, Bethesda, Maryland 20894, USA.
Tel.: +1 (301) 435-5910;
Fax: +1 (301) 435-7793;
e-mail: mygalperin@gmail.com

Cover Image: Maple Ferryman/Shutterstock.com

### EDITORIAL BOARD

Martin Ackermann, Switzerland
Sonja-Verena Albers, Germany
Mads Albertsen, Denmark
Andrew Allen, USA
Rudolf Amann, Germany
Ricardo Amils, Spain
Amy Apprill, USA
Manfred Auer, USA
Frank Aylward, USA
Mark Bailey, UK
Doug Bartlett, USA
Fernando Baquero, Spain
Ed Bayer, Israel
Oded Beja, Israel
Stefan Bertilsson, Sweden
Kaye Bidle, USA
Antje Boetius, Germany
Christophe Bordi, France
Yan Boucher, Canada
David Bourne, Australia
Mya Breitbart, USA
Erhard Bremer, Germany
Andreas Brune, Germany
Carmen Buchrieser, France
Dan Buckley, USA
Davide Bulgarelli, UK
Francesca Cardinale, Italy
Emilio Casamayor, Spain
Rick Cavicchioli, Australia
Thomas Clavel, Germany
Ralf Conrad, Germany
Pierre Cornelis, Belgium
Milton da Costa, Portugal
Daniele Daffonchio, Italy
Holger Daims, Austria
Roberto Danovaro, Italy
Eduardo Diaz, Spain
Greg Dick, USA
Elke Dittmann, Germany
Ulrich Dobrindt, Germany
Angela Douglas, USA
Nicole Dubilier, Germany
Sonya Dyhrman, USA
Leo Eberl, Switzerland
Virginia Edgcomb, USA
Ed J. Feil, UK
Patrick Forterre, France
Jed Fuhrman, USA
Geoffrey Michael Gadd, UK
Elke Genersch, Germany
Sean Michael Gibbons, USA
Steve Giovannoni, USA
Peter Girguis, USA
Lone Gram, Denmark
Hans-Peter Grossart, Germany
Susanne Häußler, Germany
Steven Hallam, Canada
John E. Hallsworth, Northern Ireland
Shige Harayama, Japan
Ian Head, UK
Michael Hecker, Germany
Tori M. Hoehler, USA
Andrew Holmes, Australia
Steve D'Hondt, USA
Matthias Horn, Austria
Phil Hugenholtz, Australia
Dana Hunt, USA
Dieter Jahn, Germany
Aaron Kaplan, Israel
David Karl, USA
Boran Kartal, The Netherlands
Nancy Keller, USA
Libusha Kelly, USA
Staffan Kjelleberg, Australia
Rob Knight, USA
Kostas Konstantinidis, USA
George Kowalchuk, The Netherlands
Michael Kühl, Denmark
Nikos C. Kyrpides, USA
Rup Lal, India
Silke Langenheder, Sweden
Jared R. Leadbetter, USA
Frédérique Le Roux, France
Debbie Lindell, Israel
Steven Lindow, USA
Karen Lloyd, USA
Frank Löffler, USA
Victor de Lorenzo, Spain
Connie Lovejoy, Canada
Derek Lovley, USA
Alexander Loy, Austria
Mike Madigan, USA
Adam Martiny, USA
Jennifer Martiny, USA
Roland Marmeisse, France
Ramon Massana, Spain
Miguel Matilla, Spain
Timothy R. McDermott, USA
Terry McGenity, UK
Mike McInerney, USA
Rainer U. Meckenstock, Germany
Itzhak Mizrahi (Itzik), Israel
Jan Roelof van der Meer, Switzerland
Gabriella Molinari, Germany
Mary Ann Moran, USA
Andrés Moya, Spain
J. Colin Murrell, UK
Ken Nealson, CA
Craig Nelson, USA
Doug Nelson, USA
Josh Neufeld, Canada
Irene Newton, USA
Graeme Nicol, UK
Per Halkjaer Nielsen, Denmark
Balbina Nogales, Spain
Maarja Öpik, Estonia
Huub Op den Camp, The Netherlands
Victoria Orphan, USA
William Orsi, Germany
Jörg Overmann, Germany
Norman R. Pace, USA
Hans Paerl, USA
Matthew Parsek, USA
Jakob Pernthaler, Switzerland
Jennifer Pett-Ridge, USA
Jim Prosser, Aberdeen, UK
Carla Pruzzo, Italy
Pei-Yuan Qian, China
Christopher Quince, UK
Ralf Rabus, Germany
Paul Rainey, New Zealand
Niels Peter Revsbech, Denmark
Thomas Richards, UK
Katharina Riedel, Germany
Ute Römling, Sweden
Forest Rohwer, USA
Fernando Rojo, Spain
Michael Rother, Germany
Mak Saito, USA

environmental
microbiology reports

**EDITORIAL BOARD cont.**

Juan Sanjuán, Spain
Karin Sauer, USA
Bernhard Schink, Germany
Tom Schmidt, USA
Andreas Schramm, Denmark
Dirk Schüler, Germany
Angela Sessitsch, Austria
Justin Seymour, Australia
Ashley Shade, USA
Brajesh Singh, Australia
Mitch Sogin, USA
Lisa Y. Stein, Canada
Thorsten Stoeck, Germany
Matthew B. Sullivan, USA
Mike Taylor, New Zealand
Andreas Teske, USA
Bo Thamdrup, Denmark
Torsten Thomas, Australia
Linda Thomashow, USA
Burkhard Tümmler, Germany
Massimo Turina, Italy
Gene Tyson, Australia
Gottfried Unden, Germany
Tim Urich, Austria
Marcel van der Heijden, Switzerland
Miguel Vicente, Spain
Christian Voolstra, Saudi Arabia
Julia A. Vorholt, Switzerland
Willem M. de Vos, The Netherlands
Larry Wackett, USA
Judy Wall, USA
David Walsh, Canada
Eric A. Webb, USA
Paul Williams, UK
Patrick Wincker, France
Benjamin Wolfe, USA
K. Eric Wommack, USA
Daniel Wozniak, USA
Kelly Wrighton, USA
JinRong Xu, USA
Michail M. Yakimov, Italy
Etienne Yergeau, Canada
Jonathan P. Zehr, USA
Steve Zinder, USA
Alga Zuccaro, Germany

### SCOPE

*Environmental Microbiology Reports (EMIR)* publishes full length research articles as well as brief reports, minireviews, opinion articles and highlights.

The scope of the Journal encompasses the diversity of current research on microbial processes in the environment, microbial communities, interactions and evolution and includes, but is not limited to, the following:

- the structure, activities and communal behaviour of microbial communities
- microbial community genetics and evolutionary processes
- microbial symbioses, microbial • interactions and interactions with plants, animals and abiotic factors
- microbes in the tree of life, microbial diversification and evolution
- microbiomes and holobionts
- population biology and clonal structure
- microbial metabolic and structural diversity
- microbial physiology, growth and survival
- primary and secondary metabolism in the environment
- microbes and surfaces, adhesion and biofilms
- responses to environmental signals and stress factors
- modelling and theory development
- pollution microbiology
- microbial ecology of pathogens
- extremophiles and life in extreme and • unusual little-explored habitats
- element cycles and biogeochemical processes
- microbes in a changing world, • microbially-influenced global changes
- systems biology and computational predictions
- evolution and diversity of archaeal and bacterial viruses
- new technological developments in • microbial ecology and evolution, in particular for the study of activities of microbial communities, non-culturable microorganisms and emerging pathogens
- single cell microbial ecology

**Open Access and Copyright**
All articles published by *Environmental Microbiology Reports* [submitted after 16th August 2022] are fully open access: immediately freely available to read, download, and share. All articles [accepted from 16th August 2022] are published under the terms of a Creative Commons license. Copyright on any research article published by *Environmental Microbiology Reports* is retained by the author(s).
Further information about open access licenses and copyright can be found at: https://authorservices.wiley.com/open-research/open-access/index.html. Information on the licenses used by *Environmental Microbiology Reports* can be found at: https://sfamjournals.onlinelibrary.wiley.com/journal/17582229.

***Copyright & Copying***. Copyright © 2023 Applied Microbiology International and John Wiley & Sons Ltd. All rights reserved. No part of this publication may be reproduced, stored or transmitted in any form or by any means without the prior permission in writing from the copyright holder. Authorization to copy items for internal and personal use is granted by the copyright holder for libraries and other users registered with their local Reproduction Rights Organisation (RRO), e.g. Copyright Clearance Center (CCC), 222 Rosewood Drive, Danvers, MA 01923, USA (www.copyright.com), provided the appropriate fee is paid directly to the RRO. This consent does not extend to other kinds of copying such as copying for general distribution, for advertising or promotional purposes, for republication, for creating new collective works or for resale. Permissions for such reuse can be obtained using the RightsLink “Request Permissions” link on Wiley Online Library. Special requests should be addressed to: permissions@wiley.com

***Disclaimer.*** The Publisher, Applied Microbiology International and Editors cannot be held responsible for errors or any consequences arising from the use of information contained in this journal; the views and opinions expressed do not necessarily reflect those of the Publisher, Applied Microbiology International and Editors; neither does the publication of advertisements constitute any endorsement by the Publisher, Applied Microbiology International and Editors of the products advertised.

Wiley Open Access articles posted to repositories or websites are without warranty from Wiley of any kind, either express or implied, including, but not limited to, warranties of merchantability, fitness for a particular purpose, or non-infringement. To the fullest extent permitted by law Wiley disclaims all liability for any loss or damage arising out of, or in connection, with the use of or inability to use the content.
